# UV Hyperspectral Imaging as Process Analytical Tool for the Characterization of Oxide Layers and Copper States on Direct Bonded Copper

**DOI:** 10.3390/s21217332

**Published:** 2021-11-04

**Authors:** Mohammad Al Ktash, Mona Stefanakis, Tim Englert, Maryam S. L. Drechsel, Jan Stiedl, Simon Green, Timo Jacob, Barbara Boldrini, Edwin Ostertag, Karsten Rebner, Marc Brecht

**Affiliations:** 1 Process Analysis and Technology PA & T, Reutlingen University, Alteburgstraße 150, 72762 Reutlingen, Germany; mohammad.alktash@reutlingen-university.de (M.A.K.); Mona.Stefanakis@reutlingen-university.de (M.S.); maryam_sanaz_lisa.drechsel@student.reutlingen-university.de (M.S.L.D.); Barbara.Boldrini@reutlingen-university.de (B.B.); Edwin.Ostertag@reutlingen-university.de (E.O.); karsten.rebner@reutlingen-university.de (K.R.); 2 Institute of Physical and Theoretical Chemistry, Eberhard Karls University Tübingen, Auf der Morgenstelle 18, 72076 Tübingen, Germany; 3 Robert Bosch GmbH, Automotive Electronics, Postfach 1342, 72703 Reutlingen, Germany; Tim.Englert2@de.bosch.com (T.E.); Jan.Stiedl@de.bosch.com (J.S.); Simon.Green@de.bosch.com (S.G.); 4 Institute of Electrochemistry, Ulm University, Albert-Einstein-Allee 47, 89081 Ulm, Germany; timo.jacob@uni-ulm.de

**Keywords:** hyperspectral imaging, pushbroom, UV spectroscopy, principal component analysis, partial least squares regression, direct bonded copper, copper oxide layer thickness

## Abstract

Hyperspectral imaging and reflectance spectroscopy in the range from 200–380 nm were used to rapidly detect and characterize copper oxidation states and their layer thicknesses on direct bonded copper in a non-destructive way. Single-point UV reflectance spectroscopy, as a well-established method, was utilized to compare the quality of the hyperspectral imaging results. For the laterally resolved measurements of the copper surfaces an UV hyperspectral imaging setup based on a pushbroom imager was used. Six different types of direct bonded copper were studied. Each type had a different oxide layer thickness and was analyzed by depth profiling using X-ray photoelectron spectroscopy. In total, 28 samples were measured to develop multivariate models to characterize and predict the oxide layer thicknesses. The principal component analysis models (PCA) enabled a general differentiation between the sample types on the first two PCs with 100.0% and 96% explained variance for UV spectroscopy and hyperspectral imaging, respectively. Partial least squares regression (PLS-R) models showed reliable performance with *R*^2^_c_ = 0.94 and 0.94 and RMSEC = 1.64 nm and 1.76 nm, respectively. The developed in-line prototype system combined with multivariate data modeling shows high potential for further development of this technique towards real large-scale processes.

## 1. Introduction

Copper is considered as one of the most important conductors for integrated circuit (IC) packaging and wire bonding. It has significant advantages in comparison to other materials (e.g., aluminum) and is thus a good alternative for smaller structures. Copper as a metal has a high mechanical stability and excellent electrical and thermal conductivities at low cost [1]. However, copper contact surfaces contaminate and interact with oxygen to copper (I) oxide (Cu_2_O) and copper (II) oxide (CuO) layers. This process is considered a problem as it influences the conductivity efficiency. Science and engineering progress has driven the development of sensor technology in the past years [2,3]. This led to novel optical sensors, such as hyperspectral imagers, to identify quality problems [4,5].

Hyperspectral imaging is a technique that integrates a conventional spectroscopic system with imaging in order to acquire spectral and spatial information from the area of interest [6,7,8,9]. Therefore, hyperspectral imaging enables quantitative analysis with improved levels of accuracy [4,10,11]. It is considered as a rapid, non-destructive and robust method. Combining spectral imaging with chemometric algorithms opens up new industrial applications, including manufacturing process control [12]. Such spectral imaging systems are used in different fields, such as food, pharmaceutical and textile production, as well as agriculture, military, astronomy, life sciences and medicine [4,5,13,14,15,16].

Hyperspectral imaging is able to capture images in different spectral bands, such as in the visible (Vis), infrared (NIR) and ultraviolet (UV) range. In contrast, traditional methods, such as Auger electron and X-ray photoelectron spectroscopy (XPS), which are used to analyze copper samples, are time consuming, expensive and require sample preparation and destruction [17,18]. The industry demands a high lateral resolution, which cannot be fulfilled by single-point UV-Vis spectroscopy [11,19]. Several detection methods have been developed to classify and identify the copper state and copper oxide layers. In the past, UV-Vis/NIR spectroscopic applications as well as Vis/NIR hyperspectral imaging have been preferred in the industrial environment, especially for copper and other metal conductors [16,17,18,19,20,21]. The detection and characterization of oxide layers on metallic copper samples was studied by Stiedl et al. using visible hyperspectral imaging and UV-Vis spectroscopy. They were able to detect the thickness of the oxide layers on the technical copper [16,17].

Recently, Tschannerl et al. have shown the application of hyperspectral imaging in the UV range to discriminate between phenolic flavor concentrations in melted barley [7]. In another recently published study, Al Ktash et al. have developed this technology in the direction of real applications. The authors were able to precisely classify between different active pharmaceutical ingredients (API) and painkiller tablets by using an UV hyperspectral imaging prototype [11].

Hyperspectral imaging collects information in three dimensions (x, y, λ), resulting in a massive number of variables. Therefore, data reduction algorithms, such as principal component analysis (PCA) and partial least squares regression (PLS-R), are required. PCA combined with hyperspectral imaging data enables the detection of spectral features in the spectroscopic data along with identifying the relative distribution of the components in mixtures [22,23]. The PLS-R is an empirical data-driven modelling approach that relies on representative model building data for two variable blocks (X and Y). It is used to search for a correlation between a simple and easily acquirable data set (X) and a labor- as well as cost-intensive second set of measurements (Y) by calculating a certain number of factors. In the present study, the X data contains the UV spectra, and the Y data the oxide layer thickness of the direct bonded copper sheets. Consequently, quantitative descriptions and calibrations are possible [24].

Despite several studies having focused on the characterization of copper oxide films, sample homogeneity remains a big challenge in the estimation of their thicknesses over the complete surface. We address this topic in the present contribution using a hyperspectral imaging system in the UV wavelength range for the in-line characterization of copper states and oxide layers thicknesses on direct bonded copper. The data were evaluated by PCA and PLS-R. The results show that hyperspectral imaging in the UV range has the potential to predict oxide layer thicknesses and copper states in a rapid and non-destructive manner.

## 2. Materials and Methods

### 2.1. Samples

**Figure 1:**
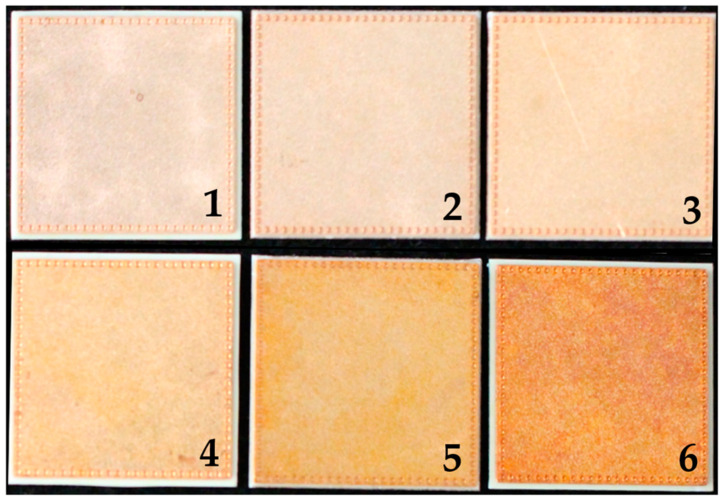
Direct bonded copper Curamik^®^Power substrates. (**1**) is an example of sample type 1, (**2**) sample type 2, (**3**) sample type 3, (**4**) sample type 4, (**5**) sample type 5 and (**6**) sample type 6.

In total, 28 direct bonded copper Curamik^®^ Power substrates (Rogers Corporation, Chandler, AZ, USA) with dimensions of 21.0 mm × 21.0 mm × 1.1 mm were used for sample preparations. The samples were first ultrasonically cleaned at 50 °C for 5 min with Vigon A 200 (Zestron, Ingolstadt, Germany) as cleaning medium and then rinsed with deionized water for 3 min. The copper sheets were oxidized at five different preparation protocols (see Table 1). Sample type 1 was left in its initial condition. Figure 1 shows an example of each copper sheet type.

### 2.2. Oxide Layer Thickness Measurement

The thicknesses of the oxide layers were determined by depth profiling using X-ray photoelectron spectroscopy (XPS). The measurements were conducted under a system base pressure of 4.0 × 10^−10^ mbar. A monochromatic Al Kα radiation was used and the anode tube operated at 12.5 kV with 20 mA. The take-off angle for the electrons was 0° with respect to the surface normal. The XPS core level spectra were measured with a standard X-ray source SPECS XR50 (SPECS Surface Nano Analysis GmbH, Berlin, Germany) and a concentric hemispherical analyzer Phoibos 100, SPECS (SPECS Surface Nano Analysis GmbH, Berlin, Germany). The pass energy of the concentric hemispherical analyzer was 50 eV for the survey and 20 eV for the high-resolution spectra. The data acquisition was performed with 0.5 eV; 0.1 eV per step, respectively.

### 2.3. UV Spectroscopy

**Figure 2:**
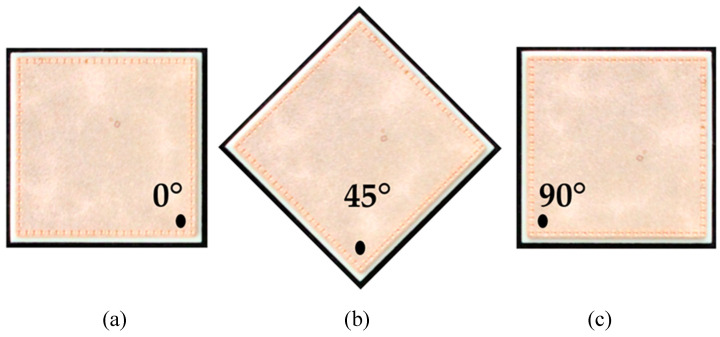
An example of a direct bonded copper sheet rotated according to the three different measurement angles (**a**) 0°, (**b**) 45° and (**c**) 90°.

Total (specular and diffuse) reflectance spectra were recorded in the range of 200–380 nm using a UV spectrometer (Lambda 1050+, PerkinElmer, Inc., Waltham, MA, USA). The 150 mm integrating sphere module functioned as a detection unit and was deployed in reflectance with a R6872-Photomultiplier (PMT). A deuterium lamp was used as light source in the spectrometer. The samples were placed at the reflectance port of the integrating sphere with a diffused scattering Spectralon^®^ disk placed behind the samples. The port measuring area is approximately 0.42 cm^2^. Three spectra were recorded for each direct bonded copper type while the sample was rotated in different angles (see Figure 2). The UV spectra were recorded with the Lambda 1050 UV WinLab software from PerkinElmer.

### 2.4. Data Collection and Preprocessing

The hyperspectral imaging setup was optimized compared to our previous work [11]. The pushbroom imager is a BlueEye Tec (inno-spec GmbH, Nürnberg, Germany), consisting of a spectrograph (RS 50–1938, inno-spec GmbH, Nürnberg, Germany), with a slit width of 80 µm, connected to a back-illuminated CMOS camera with total size 2048 × 2048 pixel (spatial × spectral) and pixel size of 6.5 µm × 6.5 µm. Additionally, the dispersion is approximately 0.1 nm/px [25]. The quantum efficiency of the CMOS camera is between 30 and 50% [26]. The optimal integration time was 10 ms. The samples were placed on a black conveyor belt (700 mm × 215 mm × 60 mm, Dobot Magician, Shenzhen Yuejiang Technology Co., Ltd., Shenzhen, China) moving with a constant speed of 0.15 mm/s, which was positioned completely in a tunnel made of PTFE. The illumination was provided by two ozone producing Xenon lamps (XBO, 14 V, 75 W, OSRAM, München, Germany). The ozone was eliminated by a laboratory vacuum system (AirTracker, TEKA Absaug- und Entsorgungstechnologie GmbH, Coesfeld, Germany). Another xenon lamp was added to the setup to increase the intensity and optimize the integration time. In combination with the black conveyor belt and a state-of-the-art UV pushbroom imager a more industrial-like prototype was created.

The principal and workflow of the data acquisition remained [11]. The UV hyperspectral imaging data were acquired by the FluxRecorder version 4.2.1.17 (inno-spec GmbH, Nürnberg, Germany). The reflectance was calculated by the FluxRecorder automatically according to the radiometric calibration [6,8,11,27]. PTFE was used as white reference. For collecting the dark reference, the objective was closed by its cover and the illumination was turned off.

**Figure 3:**
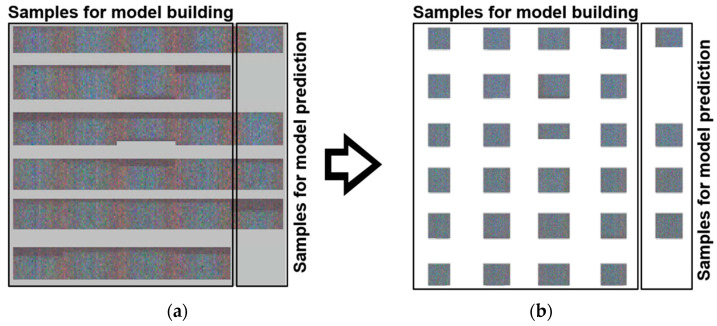
Hyperspectral raw images of 28 direct bonded copper samples on the left (**a**). Images after subtraction of the background on the right (**b**). In total, 24 samples were used for building the PLS-R model and four samples were used for prediction.

Figure 3 shows the original images of the direct bonded copper samples before and after background subtraction. Hyperspectral data matrices were analyzed by Evince version 2.7.11 (Prediktera AB, Umeå, Sweden). While importing the raw data in Evince, a data reduction was performed by binning four columns and rows (x, y) and six channels (λ).

The background was removed by calculating a PCA and selecting the corresponding background scores. Therefore, some edges and borders of the samples were also eliminated, resulting in different sample shapes (Figure 3). The reduced hypercube was then used as input for the subsequent PCA and PLS-R. In the end, approximately 2.0 million spectra remained from the initially obtained 4.0 million spectra.

### 2.5. Multivariate Data Analysis and Data Handling

Multivariate data analysis (MVA) was performed with “The Unscrambler X 10.5” (Camo Analytics AS, Oslo, Norway). All spectra recorded by UV hyperspectral imaging and commercial spectroscopy were preprocessed in the same way: Gaussian smoothing with 15 points reduction in the range from 200 nm to 380 nm. The spectral resolution of the hyperspectral imaging data was further reduced to 1 nm by averaging to ensure comparability to the UV spectra of the single-point spectrometer. The principal component analysis (PCA) was calculated with mean centering, cross-validation and the NIPALS algorithm to distinguish between the direct bonded copper sample types.

Partial least square regression (PLS-R) models for the oxide layer thickness prediction were created with mean centering, full cross-validation and the Kernel algorithm. Four direct bonded copper sheets of each preparation type were used to develop the PLS-R model. Additionally, the remaining samples of copper type 1, 3, 4 and 5 were used as prediction samples to test the final PLS-R model. The predicted values were compared to the determined oxide layer thicknesses by XPS. Finally, the oxide layer thickness of each pixel of the remaining samples was predicted by the hyperspectral imaging PLS-R model. The distribution map thus generated was visualized by MATLAB (R2020b 9.9.0, Mathworks, Natick, MA, USA). The samples were binned by factor 5 in the x and y direction due to the large amount of data and noise.

## 3. Results and Discussion

### 3.1. UV Spectroscopy

**Figure 4:**
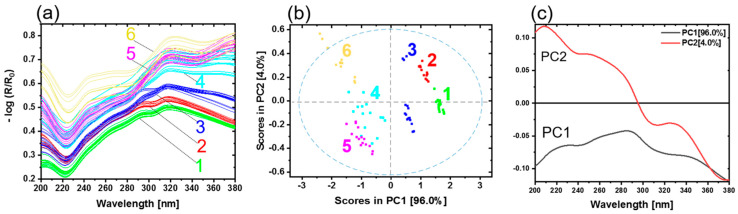
(**a**) UV reflectance spectra of copper sheets. Copper with initial condition type 1 (green), 2 (red), 3 (blue), 4 (light blue), 5 (pink) and 6 (yellow) represent the oxidation layer thicknesses 0 nm, 4 nm, 8.3 nm, 14 nm and 21.1 nm, respectively. (**b**) PCA with scores and (**c**) the corresponding loadings plot.

Direct bonded copper substrates were investigated using diffuse reflectance spectroscopy in the UV region (200–380 nm). In total, 28 samples were measured. Generally, the thickness of the oxide layers increases with the oxidation time and temperature. During the oxidation process, copper is oxidized first to copper (I) oxide (CuO_2_) and then to copper (II) oxide (CuO). Figure 4a shows the preprocessed reflectance spectra. Based on the shape of the spectra, the different steps of the oxidation process can be observed. Sample type 1 is representing copper in its initial condition. The other samples have undergone an oxidation process, as detailed in Table 1. A band minimum is detected approximately at 220 nm. A pronounced band maximum for all copper samples occurs in the wavelength range from 315 to 320 nm. Weak shoulders at 243 nm (sh) and 266 nm (sh) are observed. Sample types 1, 2 and 3 present one prominent maximum at 295 nm. Sample types 4, 5 and 6 show a distinct band with maximum at 378 nm. The band at 220 nm could be ascribed to Cu_2_O. Increasing Cu_2_O pronounces the minimum. The band at 295 nm is assigned to the copper material (see Supplementary Figure S1). This band started to fade away due to the increase in the maximum band at 320 nm. This band is absent in sample types 4, 5 and 6. For these sample types a band at 378 nm appears. These spectral differences were due to different oxide layer thicknesses and copper states (Cu^0^, Cu_2_O and CuO) on the copper sheets. The remaining small differences among the spectra were attributed to the roughness, measuring angles and sample positions.

Figure 4b shows the scores plot of the first two principal components (PC). The first two PCs explain nearly 100.0% of the total variance. The scores of different sample types are clearly distinguished. Every copper sample type with a corresponding copper state and oxide layer thickness appear as a distinct group. PC1 yields a clear separation of copper in the initial condition (type 1) from the other copper types. The groups move below the average in PC1, with increasing oxide layer thickness and conversion of copper states. Copper types 4 and 5 are slightly overlapped as their oxide layer thicknesses are almost comparable (see Table 1). The variance in each cluster results from the different samples for each type. The differences between the samples could be due to temperature profiles in the oven while preparing the samples, roughness variation, or sample positioning during the measurements.

The loadings plot for PC1 and PC2 is given in Figure 4c. The shape of PC1 resembles the Cu^0^ spectrum (see Figure S1 and Table S1). This indicates that an increasing amount of Cu^0^ on a sample results in a more positive sample arrangement on PC1. Vice versa, the less Cu^0^ is present in the samples because of the growing oxide layer thickness, the more the samples are shifted in the negative range of PC1. The influence of the oxidation state (Cu_2_O, CuO) is expressed by PC2 (see Figure S1 and Table S1); these results are comparable with previous studies [16].

### 3.2. UV Hyperspectral Imaging

**Figure 5:**
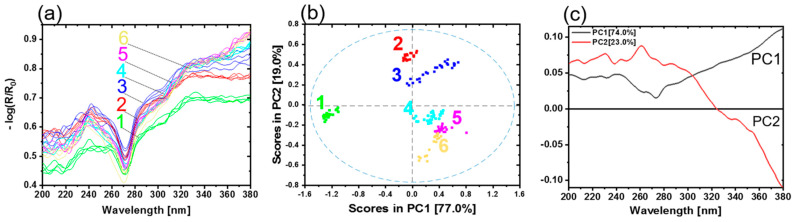
(**a**) Average UV hyperspectral imaging spectra of copper sheets. Copper with initial condition type 1 (green), 2 (red), 3 (blue), 4 (light blue), 5 (pink) and 6 (yellow) represent the oxidation layer thicknesses 0 nm, 4 nm, 8.3 nm, 14 nm and 21.1 nm, respectively. (**b**) PCA with scores and (**c**) the corresponding loadings.

All samples were analyzed by a UV hyperspectral imaging prototype, as described in Materials and Methods. In order to make the data more comparable to the UV spectroscopy, the average spectra were calculated to reduce the number of spectra. A total of 25 spectra was determined from the hyperspectral imaging data for each of the 28 samples. Figure 5a shows the results of the UV hyperspectral imaging in the range from 200 to 380 nm.

The comparison between the shapes of the spectra is given in Figure 4a and Figure 5a, showing similarities as well as a small deviation. They are due to the type of the illumination source and the design of the experimental setups. For reflectance spectroscopy, a deuterium lamp was used, while for hyperspectral imaging, two xenon lamps were available. Deuterium lamps have higher spectral irradiances in the deep UV range compared to xenon lamps [28]. However, the xenon illumination was sufficient for the characterization of direct bonded copper sheets. Therefore, the interferences < 270 nm are more pronounced compared to the higher wavelengths. As a result, the spectra shown in Figure 5a provide almost no clearly recognizable spectroscopic information in the region <270 nm. The detector’s efficiency and illumination provide low performance in this wavelength range. Therefore, the easily accessible tunnel design for hyperspectral imaging was developed to ensure a diffuse illumination of the samples. As a result, a reasonable illumination strength and homogeneity were reached.

As discussed before, the spectra were influenced by the copper states and thicknesses of the oxide layers on the copper sheets. Copper in the initial condition is represented in the spectra originating from sample type 1 (see Table 1, Figure 4a). The most dominant contributions for all copper sample types are observed in the wavelength range 324–328 nm and at 241 nm. Copper types 1, 2 and 3 present a weak shoulder at 292 nm.

In the next step, a PCA model with a cross-validation was calculated for the average spectra of all samples. Figure 5b shows the scores plot of PC1 and PC2. The first two PCs explain nearly 96.0% of the total variance. The scores of different sample types are clearly distinguished. Every copper sample type with a corresponding copper state and oxide layer thickness appears as a distinct group. PC1 yields a clear separation of copper with initial condition (type 1) from the other copper types. A discrimination of the copper state and oxide layer thickness is observed on PC2. Beginning from the positive to the negative scores on PC2, the samples are arranged in the order copper type 2, 3, 4, 5 and 6, respectively. Again, copper type 2 and 3 (positive scores) can be separated from the other samples 4, 5 and 6 (negative scores).

The loadings plot for PC1 and PC2 is given in Figure 5c. PC1 shows the differences between Cu^0^ and the oxidation states (Cu_2_O, CuO). The most dominant contribution is observed in the range from 260 to 280 nm and the increasing shape > 280 nm. The loadings plot of PC2 mainly shows increasing oxide layer thickness. The most prominent contribution is observed in the range from 250 to 280 nm and the decreasing shape > 280 nm. Compared to PC1, PC2 has a positive maximum at 263 nm. The minimum on PC1 is located at 273 nm. This region could include the information about the copper state. The influence of the oxidation state (Cu_2_O, CuO) and oxide layer thickness is observed by PC2.

Figure 4a presents UV spectra with a good signal-to-noise-ratio recorded by a UV spectrometer, which collected one single spectrum over an area of 0.42 cm^2^. Figure 5a shows the UV spectra recorded by the hyperspectral imaging setup. The spectra were averaged over an area size comparable to the UV spectrometer. The UV hyperspectral imager recorded raw spectra with a less good signal-to-noise-ratio. These spectra result from one single pixel of the detector, representing a much smaller area of the direct bonded copper, which is estimated to be 6.5 µm × 6.5 µm. Additional reasons for the low signal-to-noise-ratio are the weak irradiation intensity by the xenon illumination and the quantum efficiency of the camera in this spectral range of approximately 30–50% [26]. Furthermore, ozone-producing xenon lamps were used. With the help of a vacuum system, the influence of the ozone absorption at 250 nm was minimized.

**Figure 6:**
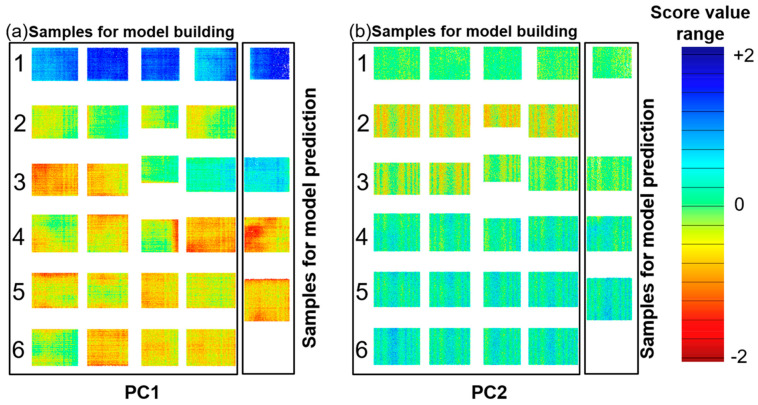
Distribution maps of the oxide layer PC1 (**a**) and PC2 (**b**). Each rectangle represents a single copper sheet. The sample type for each row corresponds to Table 1. The samples are divided into two sets: model building and model prediction for PLS-R. The colored pixels (the score value range) represent the oxide content, from low (blue) to high (red).

The benefit of hyperspectral imaging is lateral information in real time. To get a visual impression of the inhomogeneity of the copper states and oxide layer thickness, the thickness for every pixel from the first two PCs was plotted as a distribution map, shown in Figure 6. A sample with high absorbance has a high proportion of blue in the score image (e.g., Cu^0^), while one with low absorbance shows a higher proportion of red (e.g., Cu(II)). Clear differences between the samples are observed according to the oxidation time and temperature. As discussed before, PC1 yields a clear separation of copper in the initial condition from the other copper types. A discrimination of the copper state and oxide layer thickness can be observed on PC2. The regular distribution of the pattern in PC2 indicates a common origin; this could be the variability of the temperature inside the oven among each sample. Additionally, in the distribution maps, it is possible to clearly identify oxidation hotspots on the direct bonded copper.

### 3.3. PLS-R

A PCA structures data sets according to their maximum variance, whereas PLS-R searches for the optimal correlation between spectral characteristics and an external target value. PLS-R models of the direct bonded copper for each method have been established and compared, by using the spectra of the UV reflectance spectroscopy and UV hyperspectral imaging. In this study, spectral features were extracted from the spectral datasets and correlated to determine the oxide layer thickness via XPS.

Gaussian smoothing with 15 points was performed to minimize the noise. A PLS-R model was developed with a calibration set of *n* = 24 samples (see Figure 6), three factors, the Kernel algorithm and full cross-validation. A prediction sample set was used to test the PLS-R model performance with an external validation to assess the predictive ability. The prediction sample set consisted of four samples with mean oxide layer thicknesses of 0 nm, 6 nm, 8.3 nm and 14 nm (see Figure 6). Table 2 summarizes the overall chemometric model results for both the UV spectroscopy and hyperspectral imaging.

The number of factors for each PLS-R model was optimized according to a high coefficient of determination (*R*^2^) and a low root mean square error of calibration (RMSEC) and cross-validation (RMSECV). This approach was applied to both the calibration (*R*^2^_c_) and cross-validation (*R*^2^_cv_) model for each method (Table 2).

The variances explained by the UV reflectance model for the X and Y variables were 99.0% and 95.0%, respectively, by using three factors. The variances of the X and Y variables were 98% and 94% for the UV hyperspectral imaging model, by using three factors as well. This indicated that three PLS components (factors) were sufficient to describe most of the variance in the data according to the spectral information.

**Figure 7:**
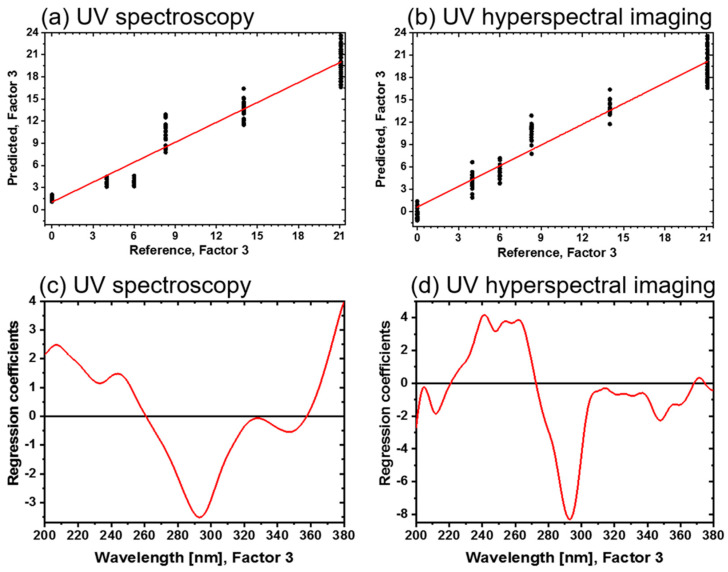
Three-factor PLS-R models for the oxide layer thicknesses of direct bonded copper in the UV region (200–380 nm). (**a**) Predicted vs. reference of UV spectra. (**b**) Predicted vs. reference of UV hyperspectral imaging. (**c**) Regression coefficients of the UV spectra. (**d**) Regression coefficients of the UV hyperspectral imaging.

The results show that the PLS-R models are very effective in correlating the oxide layer thickness with both spectroscopic data sets. This is indicated by a high *R*^2^_c_ and a low RMSEC and a high *R*^2^_cv_ with a low RMSECV (see Table 2). Figure 7a,b show the correlation between the reference and predicted values of the UV reflectance spectra and UV hyperspectral imaging, respectively. The deviation and the variance within a sample type are increasing according to the oxide layer growth on the direct bonded copper. Copper sample types 1, 2 and 3 have a smaller variance within the sample type. In contrast, sample types 4, 5 and 6 have more variance in the UV reflectance spectra model. For UV hyperspectral imaging all samples have nearly the same variance. This variance is probably due to the efficiency of the detector and the illumination in both setups.

The regression coefficients of the three-factor UV spectroscopy PLS-R model are shown in Figure 7c. Again, absorbance bands around 210 nm, 245 nm, 293 nm and 330 nm emerge, as displayed in the spectra. Above 360 nm, an increasing baseline in the regression coefficient plot is registered. In Figure 7d, the corresponding regression coefficients of the UV hyperspectral imaging PLS-R model are displayed. They have a comparable shape, but more details can be detected. For example, in the range <260 nm and from 310 to 340 nm, more spectral features are pronounced. At 370 nm, a defined band appears for the UV hyperspectral imaging model, while an increase >360 nm in the UV spectroscopy model is registered.

Correlated to the bands at <260 nm, 320 nm, 335 nm and >360 nm, the oxide layer thickness increases in the UV spectra, which is also comparable to UV hyperspectral imaging in the range 230–265 nm and 306–340 nm for increasing the oxide layer thickness. At 293 nm, the oxide layer thickness decreases for both setups. Differences in the beginning and ending of the regression coefficients between both methods could be due to the detectors limits with the UV hyperspectral imaging setup, as already discussed in the literature [11,16].

In order to evaluate the PLS-R models, four samples of type 1, 3, 4 and 5 were used to test the model’s performance by predicting the oxide layer thickness. The sample set contains four samples with mean oxide layer thicknesses of 0 nm, 6 nm, 8.3 nm and 14 nm (see Figure 6).

In addition, the results indicated that the PLS-R was very effective in predicting the oxide layer thickness with three factors, *R*^2^_p_ = 0.90 with RMSEP = 1.62 nm and bias = 0.51 for UV spectra, and *R*^2^_p_ = 0.85 with RMSEP = 1.98 nm and bias = 0.61 for UV hyperspectral imaging.

In Table 3, the results for the mean value of the predictions and deviations are given. The predicted values are matched well with the references.

**Figure 8:**
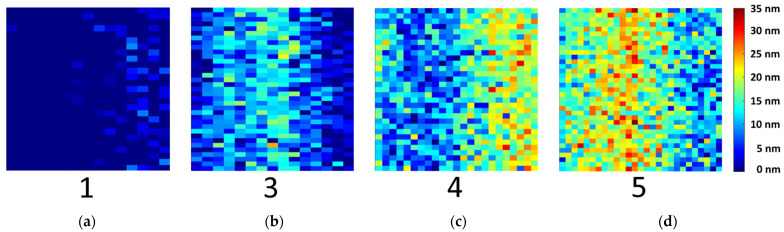
Distribution map predicted from the three-factor PLS-R model of the UV hyperspectral imaging data. The oxide layer thicknesses for each pixel of samples (**a**) sample type 1, (**b**) sample type 3, (**c**) sample type 4 and (**d**) sample type 5 were calculated for model prediction.

Mean values with a high standard deviation were measured by XPS (see Table 1), as reference values for the PLS-R models. This average of one sample type is comparable to the UV spectra recording. However, hyperspectral imaging enables to recognize different oxide layer thicknesses among the samples. Therefore, the oxide layer thickness in nm of each pixel was calculated. The UV hyperspectral imaging PLS-R model was applied to the four prediction samples of type 1, 3, 4 and 5. In Figure 8, the resulting distribution map is shown. The pixels represent the oxide layer thicknesses in nm, from low (blue) to high (red). Sample type 1 displays the initial direct bonded copper sheet without induced oxidation, while the other samples show an increasing oxide layer thickness.

Although, PLS-R is a robust model to describe the majority of the variance of the data according to the spectral information. Compared to the results of the Vis hyperspectral imaging [16], the UV hyperspectral imaging models seems to be more robust. This is indicated by the fact that less factors are necessary to achieve a model with better statistic parameters (higher *R*^2^, lower RMSE) by using a new UV prototype.

Hyperspectral imaging in the UV range is rarely reported, although it is often chosen for process control and quality assurance [7,11]. The aim of this study was to characterize the copper states and oxide layer thicknesses by using a single-point UV spectrometer and a UV hyperspectral imaging setup that can serve as an example for a possible real-time industrial application. With our hyperspectral imaging prototype, a whole direct bonded copper sheet can be measured and processed within 10 s. With the implemented pushbroom imager, hardware binning is also possible, and can decrease the measuring and processing time. The scan speed for the determination of the oxide layer thicknesses on direct bonded copper can be optimized by selecting a few relevant variables instead of the complete UV spectrum. The intensity and type of the illumination are the limiting factors towards a setup for a production environment. This study opens a novel possibility for further development of this method capable of rapid in-line data acquisition, process control and in-line classification/sorting, which meets the requirements of a real-time process with industrial standard and precision.

## 4. Conclusions

UV hyperspectral imaging and UV reflectance spectroscopy (200–380 nm) were used to characterize 28 direct bonded copper samples. UV reflectance spectroscopy, as a well-known method, was utilized to compare the quality of the UV hyperspectral imaging results.

Hyperspectral imaging in combination with PCA and PLS-R is a promising approach for the laterally resolved detection and differentiation of copper states and the determination of oxide layer thickness in the UV region. The PCA models were able to separate all direct bonded copper types according to the copper states and oxide layer thicknesses, using only the first two principal components. PLS-R models with three factors provided a high *R*^2^ and low RMSE for calibration, validation (n_cv_ = 24) and prediction (n_p_ = 4). To the best of our knowledge, this is the first work reporting the identification and quantification of copper oxide thin films by UV hyperspectral imaging. The advantage of the home-built setup is the high spatial and spectral resolution and a relatively high data acquisition speed under laboratory conditions. Starting from the presented design and data given in this contribution a setup fulfilling the requirements of a real industrial process can be easily realized.

## Figures and Tables

**Table 1:** Sample preparation protocol for the direct bonded copper substrates.

Sample Type	1	2	3	4	5	6
Number of measured samples	5 *	4	5 *	5 *	5 *	4
Temperature/°C	NA	110.0	142.5	142.5	175.0	175.0
Time/min	NA	2	11	20	11	20
Mean oxide layer thickness/nm	0	4.0	6.0	8.3	14.0	21.1
Standard deviation oxide layer thickness/nm	0	5.9	3.0	4.5	7.0	8.2

* One of each sample set was used for PLS-R prediction.

**Table 2:** Model statistics for the calibration and full cross-validation models for oxide layer thickness on the direct bonded copper.

Method	Number of Factors	Parameters Calibration		Parameters Validation	
		*R* ^2^_c_	RMSEC/nm	*R* ^2^_cv_	RMSECV/nm
UV spectroscopy	3	0.94	1.64	0.93	1.74
UV hyperspectral imaging	3	0.94	1.76	0.93	1.88

**Table 3:** Prediction of the oxide layer thicknesses for direct bonded copper from PLS-R models.

Method	Sample Type	Reference/nm	Predicted/nm	Deviation/nm
UV spectroscopy	1	0	1.59	0.93
	3	6	6.00	1.02
	4	8.3	7.86	1.44
	5	14	15.25	1.53
UV hyperspectral imaging	1	0	–0.87	1.49
	3	6	5.51	2.08
	4	8.3	11.74	1.91
	5	14	14.35	1.79

## Data Availability

The raw/processed data required to reproduce these findings cannot be shared at this time as the data also forms part of an ongoing Ph.D. thesis.

## Supplementary Materials

The following are available online at https://www.mdpi.com/article/10.3390/s21217332/s1, Figure S1: Reference spectra for the copper Cu^0^, Cu_2_O and CuO by using UV spectrometer, Table S1: Description of the direct bonded copper substrates and their sample preparation.
