# Supplementary material for: UV Hyperspectral Imaging as Process Analytical Tool for the Characterization of Oxide Layers and Copper States on Direct Bonded Copper
Source: Sensors (Basel). 2021 Nov 4;21(21):7332. doi: 10.3390/s21217332 (PMC8588143; doi:10.3390/s21217332)
Supplement: Supplementary file 1 [file sensors-21-07332-s001.zip › sensors-1437641-supplementary.pdf]

sensors

MDPI

Supplementary Material

UV Hyperspectral Imaging as Process Analytical Tool for the Characterization of Oxide Layers and Copper States on Direct Bonded Copper

Mohammad Al Ktash [1,2,†], Mona Stefanakis [1,2,†], Tim Englert [3,4], Maryam S. L. Drechsel [1], Jan Stiedl [3], Simon Green [3], Timo Jacob [4], Barbara Boldrini [1], Edwin Ostertag [1], Karsten Rebner [1] and Marc Brecht [1,2,*]

1 Process Analysis and Technology PA & T, Reutlingen University, Alteburgstraße 150, 72762 Reutlingen, Germany; mohammad.alktash@reutlingen-university.de (M.A.K.); Mona.Stefanakis@reutlingen-university.de (M.S.); maryam_sanaz_lisa.drechsel@student.reutlingen-university.de (M.S.L.D.); Barbara.Boldrini@reutlingen-university.de (B.B.); Edwin.Ostertag@reutlingen-university.de (E.O.); karsten.rebner@reutlingen-university.de (K.R.)
2 Institute of Physical and Theoretical Chemistry, Eberhard Karls University Tübingen, Auf der Morgenstelle 18, 72076 Tübingen, Germany
3 Robert Bosch GmbH, Automotive Electronics, Postfach 1342, 72703 Reutlingen, Germany; Tim.Englert2@de.bosch.com (T.E.); Jan.Stiedl@de.bosch.com (J.S.); Simon.Green@de.bosch.com (S.G.)
4 Institute of Electrochemistry, Ulm University, Albert-Einstein-Allee 47, 89081 Ulm, Germany; timo.jacob@uni-ulm.de
* Correspondence: Marc.Brecht@Reutlingen-University.de
† These authors contributed equally to the work.

Citation: Al Ktash, M.; Stefanakis, M.; Englert, T.; Drechsel, M. S. L.; Stiedl, J.; Green, S.; Jacob, T.; Boldrini, B.; Ostertag, E.; Rebner, K.; et al. UV Hyperspectral Imaging as Process Analytical Tool for the Characterization of Oxide Layers and Copper States on Direct Bonded Copper. *Sensors* **2021**, *21*, 7332. https://doi.org/10.3390/s21217332

Academic Editor: Manuel Graña

Received: 11 October 2021
Accepted: 3 November 2021
Published: 4 November 2021

Publisher's Note: MDPI stays neutral with regard to jurisdictional claims in published maps and institutional affiliations.

Copyright: © 2021 by the authors. Submitted for possible open access publication under the terms and conditions of the Creative Commons Attribution (CC BY) license (https://creativecommons.org/licenses/by/4.0/).

Supplementary Materials:

Diffuse reflectance spectra of the copper powders were recorded in the range of 200 nm – 380 nm using a commercial UV spectrometer (Lambda 1050+, PerkinElmer, Inc., Waltham, MA, USA). The spectrometer was equipped with a 150 mm Spectralon® integrating sphere to acquire data in reflection mode with an R6872-Photomultiplier (PMT). A deuterium lamp was used as light source in the spectrometer. A 10 mm quartz SUPRASIL® cuvette (QS, 100-10-40, Hellma, Müllheim, Germany) was used for measuring the copper powder see Table S1. The filled cuvette was placed at the reflectance port of the integrating sphere. The port measuring area is approximately 4.9 cm².

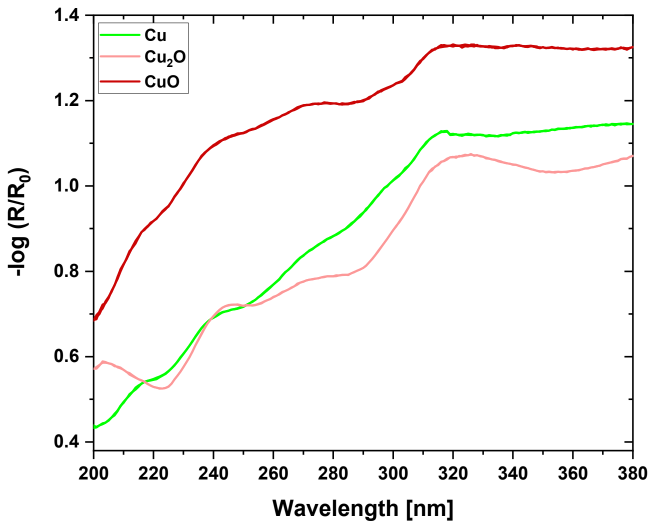

Figure S1. Reference spectra for the copper Cu^0, Cu_2O and CuO by using UV spectrometer.

Table S1. Description of the direct bonded copper substrates and their sample preparation.

Sample type	Description	Manufacturer	Article Number
Cu	Copper, powder, electrolytically produced	Merck KGaA, Darmstadt, Germany	2715
Cu2O	Copper (I) oxide powder, red	Riedel-de Haën AG, Seelze, Germany	12841
CuO	Copper (II) oxide powder, heavy, powder, technical	Riedel-de Haën AG, Seelze, Germany	12867
